# Chitosan-Based Membranes for Skin Wound Repair in a Dorsal Fold Chamber Rat Model

**DOI:** 10.3390/pharmaceutics14122736

**Published:** 2022-12-07

**Authors:** Maria Helena Casimiro, Luís M. Ferreira, Pedro M. P. Santos, João P. Leal, Gabriela Rodrigues, Inês Iria, Sara Alves, Diogo Pais, Diogo Casal

**Affiliations:** 1 Centro de Ciências e Tecnologias Nucleares (C2TN), Instituto Superior Técnico (IST), Universidade de Lisboa, 2695-066 Bobadela, Portugal; 2 Departamento de Engenharia e Ciências Nucleares (DECN), Instituto Superior Técnico (IST), Universidade de Lisboa, 2695-066 Bobadela, Portugal; 3 Centro de Química Estrutural (CQE), Instituto Superior Técnico (IST), Universidade de Lisboa, 2695-066 Bobadela, Portugal; 4 Centro de Ecologia, Evolução e Alterações Ambientais (cE3c) & CHANGE—Global Change and Sustainability Institute, Departamento de Biologia Animal, Faculdade de Ciências, Universidade de Lisboa (FCUL), 1749-016 Lisboa, Portugal; 5 Instituto de Investigação do Medicamento (iMed ULisboa), Faculdade de Farmácia, Universidade de Lisboa, 1649-003 Lisboa, Portugal; 6 Departamento de Anatomia, NOVA Medical School|Faculdade de Ciências Médicas (NMS|FCM), Universidade NOVA de Lisboa, 1169-056 Lisboa, Portugal; 7 Centro de Estudos de Doenças Crónicas (CEDOC), NOVA Medical School|Faculdade de Ciências Médicas (NMS|FCM), Universidade NOVA de Lisboa, 1150-082 Lisboa, Portugal

**Keywords:** chitosan, PVA, VP, gelatin, gamma radiation, cell proliferation, skin scaffolds, regenerative medicine

## Abstract

Frequently, deep partial and full-thickness skin wounds do not spontaneously regenerate. To restore the normal function of skin, epidermal and dermal components have to be supplied to the wound bed by grafting various substrates. Available options are limited and frequently costly. Herein, authors present a possible approach using 3D skin scaffolds capable of mimicking structure and biological functions of the extracellular matrix, providing, in parallel, a good environment for cell attachment, proliferation and differentiation. Low-molecular weight chitosan-based membranes were prepared by freeze-drying and ionizing radiation techniques to be used as skin scaffolds. Poly (vinyl alcohol), PVA, vinyl pyrrolidone, VP, and gelatin from cold water fish were incorporated. Information regarding membranes’ physical-chemical properties from SEM analysis, swelling and weight loss, together with biological response through in vitro assays (using Human Caucasian Fetal Foreskin Fibroblast) allowed the selection of an optimized batch of membranes that was used as skin scaffold in a dorsal rat model wound. The in vivo implantation assays (in Wistar rats) resulted in very promising results: (i) healing process faster than control; (ii) good vascularization; (iii) viable new tissues morphologically functional.

## 1. Introduction

The skin, which encompasses complex multilayer structures that act as a protection barrier, regulates the body temperature and provides sensation, among other important physiological functions [1].

In respect of deep partial and full-thickness skin wounds, it is known that they frequently do not spontaneously regenerate without leaving scars or other tissue alterations. Clinically, to restore normal skin function, epidermal and dermal components have to be supplied to the wound bed by grafting [2,3]. Presently, healing wounds continue to be a vexing problem in multiple cases, such as after burns, oncological resections, vascular insufficiency, diabetes, and/or radiotherapy. Available options are limited, frequently entail donor site morbidity (as in the case of autologous skin grafts or flaps) and are often costly (in the case of alloplastic reconstructive options) [4,5,6]. There is a wide range of alloplastic materials available both for research and clinical purposes, including multiple polymers and polymer composites (such as silicone, polylactic compounds, polyurethane, polycaprolactone, polytetrafluoroethylene, and high-density polyethylene), as well as naturally occurring materials, namely cellulose and silk proteins [7,8]. Despite these options, in the clinical setting, the implantation of devices composed of these materials has been associated with several complications, such as infection, extrusion, displacement, local irregularity, and hematoma formation [8]. These complications ultimately lead to the removal of the medical device in up to 28% of cases [9]. Hence, new safe and efficient alloplastic options are direly needed.

Conventional alloplastic methods used in the treatment of skin wounds provide, most of the time, a temporary cover until tissue or cell grafting can be performed or another solution provided [6,10,11]. A possible alternative is the use of 3D skin scaffolds capable of mimicking the structure and biological functions of the extracellular matrix, as well as providing a suitable environment for cells to attach, proliferate and differentiate, protecting the wound with no additional intervention [7,12].

In recent years, significant progress has been made in the development of novel biodegradable natural polymer-based materials tailored to be used as tissue scaffolds [13,14,15,16]. However, even though these polymers show good natural properties for this type of application, regardless of tissue type, a number of key considerations are important: biocompatibility, biodegradability (tuned to match the tissue regeneration rate), mechanical properties (suited to the specific application and to temporarily offer structural support until new tissue is formed), porous interconnected 3D architecture and manufacturing technology [17]. Moreover, a number of methods, such as casting [18], electrospinning [19,20] or plasma surface treatment [21] can be used to generate polymeric instructive matrices and scaffolds to be employed in tissue regeneration.

The authors’ previous work has demonstrated that 3D biocompatible and biodegradable chitosan-based matrices prepared by gamma irradiation display a very promising in vitro behavior for potential use as skin scaffolds [22,23,24], even when different composition and preparation methodologies were applied. Based on that, here the authors have extended the study to an in vivo evaluation using the developed and optimized preparation procedure, and the polymeric composition/content that has shown the most interesting in vitro results. Those previous studies have shown that the use of low-molecular weight chitosan and a dose up to 10 kGy are the key experimental parameters that lead to the most promising biological in vitro results in terms of cell growth and cell organization. Additionally, the use of radiation technology, which avoids the use of harmful initiators or solvents, is a significant improvement in the current tissue engineering field. This technology allows the tailoring of the molecular structure, surface roughness and porosity, and has already showed to be an effective route to prepare, functionalize and sterilize new materials for biomedical applications [25,26,27,28].

**Figure 1:**
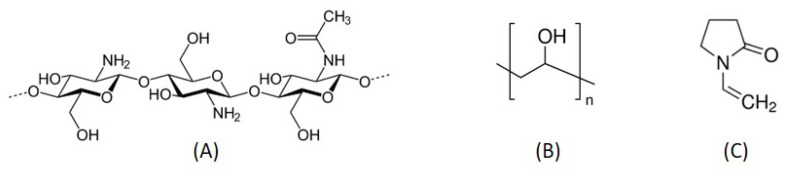
Schematic representation of: (**A**) Chitosan; (**B**) PVA; (**C**) VP.

Based on that, in the present work authors carried out a systematic study over blended/crosslinked natural and synthetic polymers prepared by gamma irradiation techniques of four selected membranes. The main polymers used were chitosan of low molecular weight (L-Chit), poly (vinyl alcohol), PVA and vinyl pyrrolidone, VP, (vd. Chemical structures at Figure 1), since they possess biological and/or mechanical properties with demonstrated interest regarding biocompatibility and skin wound healing. L-Chit, a non-toxic, biocompatible and biodegradable linear polysaccharide derived from chitin, composed of 1,4-beta-linked D-glucosamine and N-acetyl-D-glucosamine units, was the base polymer in all formulations.

Herein, a brief physical-chemical characterization of the most promising chitosan-based membranes that can improve wound healing and tissue regeneration/repair is presented (characterization by Fourier Transform Infrared Spectroscopy (FTIR) or Thermogravimetric Analysis (TGA), for instance, have already been published elsewhere [22,23]), along with the membranes’ in vitro behavior and in vivo effectiveness.

## 2. Materials and Methods

### 2.1. Materials

Low-molecular weight chitosan, L-Chit (Mw: 50,000–90,000; ≥75% deacetylated chitin), poly (vinyl alcohol), PVA (Mw: 89,000–98,000; 99+% hydrolyzed) and gelatin from cold-water fish skin (40–50% in H_2_O) were purchased from Sigma-Aldrich (Darmstadt, Germany). N-vinyl 2-pyrrolidone, VP (stabilized 98%) was obtained from Acros Organics (Geel, Belgium). Acetic acid (min 99.8%, PA) and ethanol (min 99.8%, PA) were obtained from Riedel-de-Haën (Munich, Germany).

### 2.2. Preparation of Chitosan-Based Membranes

The membranes’ preparation followed the procedure described in authors’ previous works [22]. Briefly, a 2% *m*/*v* chitosan solution in aqueous acetic acid (1% *v*/*v*) was obtained by dissolution of an appropriate chitosan amount at 40 °C with stirring, followed by filtration. Then, at room temperature, specific volumes of VP or PVA aqueous solution (10% *m*/*v*, prepared by 2 h stirring at 80 °C) were added to obtain a chitosan-based solution with 5% *m*/*v* in VP, or PVA, of the final volume. To those chitosan-based solutions, in some cases, 4% (*m*/*v*) of gelatin from cold-water fish skin was also added. Next, all solutions, L-Chit/PVA, L-Chit/PVA/Gel, L-Chit/VP and L-Chit/VP/Gel, were bubbled with N_2_, and a selected volume of each one was transferred to polystyrene Petri dishes before freezing at −26 °C overnight. Afterwards, each Petri dish was ethanol-neutralized, water-washed and finally freeze-dried. This freeze-drying procedure comprised an initial 3 h freezing at −26 °C, followed by another 3 h freezing step at −80 °C before a final 48 h lyophilization step. Hereafter, circular pieces of 10- and 12-mm diameter were cut for in vitro and in vivo essays, respectively, sealed under nitrogen atmosphere and gamma-irradiated at 10 kGy. Irradiations were performed in the cobalt-60 chamber (model Precisa 22, Graviner Lda, UK, 1971) located at the Ionizing Radiation Facilities (IRIS) from Centro de Ciências e Tecnologias Nucleares (C2TN), at a dose rate of 0.5 kGy·h^−1^. Routine Amber Perspex dosimeters from Harwell (Oxford, UK) were used for monitoring the membranes’ absorbed dose.

### 2.3. Membranes’ Characterization

#### 2.3.1. Scanning Electron Microscopy (SEM)

The morphology of the chitosan-based membranes was evaluated by SEM using a S-2400 Hitachi microscope (Tokyo, Japan) with an accelerating voltage of 20.0 kV. Prior to analysis, samples were Au/Pd coated.

#### 2.3.2. Swelling

Pre-weighted chitosan-based membranes gamma-irradiated at 10 kGy were immersed in distilled water until reaching their maximum swelling capacity (≈2 h) at room temperature. After that period, the swollen membranes were removed from distilled water and placed in another container for weighting. The swelling degree was calculated as follows:

(1) $$Swelling\;degree\;\left(\%\right)=\frac{W_{s}-W_{dry}}{W_{dry}}\times 100$$

where *W_s_* is the weight of swollen membrane and *W_d_* the weight of dried membrane.

The measurements for swelling degree determination were performed in triplicate and the results expressed as function of the mean value.

#### 2.3.3. In Vitro Degradation

To simulate membranes’ weight loss in the saline environment of the human body, previous dried and weighted chitosan-based membranes (*W*_0_) were immersed in saline solution (NaCl 0.9% *m*/*v*) at 36 °C. After 4, 7 and 21 days of immersion, samples were removed and washed with water before being air-dried at room temperature; finally, they were weighted (*W_deg_*). The extent of degradation was calculated in terms of weight loss according to Equation (2):

(2) $$Weight\;loss\;\left(\%\right)=\frac{W_{0}-W_{deg}}{W_{0}}\times 100$$

The measurements were performed in triplicate and results expressed as a function of the mean ± SD.

### 2.4. In Vitro Evaluation

In the present work, we used the commercial cell line HFFF2 (European Collection of Authenticated Cell Cultures, ECACC 86031405, Salisbury, UK), a Human Caucasian Fetal Foreskin Fibroblast cell line [22]. These cells are human dermal fibroblasts, thus representing a suitable cell model for this study. Cells were grown in Dulbecco’s Modified Eagle Medium (DMEM, Glutamax), supplemented with heat-inactivated fetal bovine serum (FBS) 10% (*v*/*v*) and streptomycin and penicillin 100 U/mL (all from Gibco, Waltham, MA, USA) at 37 °C in a humidified atmosphere with 5% of CO_2_. The culture medium was replaced every other day, and cells were used after reaching a confluence of approximately 80%.

#### Cell Viability Assay (almarBlue^®^)

In both indirect- and direct- method cell viability assays there was no need to sterilize samples since all membranes in the study were γ-irradiated in sealed bags, allowing for simultaneous preparation and sterilization. The alamarBlue^®^ reagent (purified resazurin for high sensitivity) was used to assess cell viability.

- Indirect Method

To assess a putative cytotoxic effect of the membranes on HFFF2 fibroblasts, an indirect toxicity test was performed where cells grown on a 48-well tissue culture plate were exposed to culture medium that was left in contact with several membranes for 4 days in the cell culture incubator in the above-mentioned conditions (conditioned media). An alamarBlue assay (Life Technologies, Bleiswijk, Netherlands) was performed to evaluate the viability of the cells after they were grown for 4 days in these conditioned media, as described in previous works [22,23]. The cells were incubated with 300 μL of fresh culture medium supplemented with 30 μL of alamarBlue reagent for 2 h at 37 °C in a 5% CO_2_ atmosphere. After that period, the collected medium was transferred to a 96-well plate, and the optical density (OD) was read in a microplate reader (Tecan Spectra, Männedorf, Switzerland) at 570 nm with a reference wavelength of 600 nm. Measurements made in triplicate are expressed as mean ± SD.

- Direct Method

For the direct method, cell toxicity was evaluated directly on cells growing on the membranes. γ-Irradiated sterile chitosan/PVA and PV- based membranes (ϕ 10 mm) were placed directly into 48-well tissue culture plates and prepared according to a protocol described in the authors’ previous work [22]. Briefly, a 500 μL suspension of the HFFF2 containing approximately 20,000 cells was seeded on the membranes and cultured at 37 °C. Cells growing directly on the polystyrene surface of the wells were used as control samples. After 1, 4, 7 and 14 days of culture on the membranes, cell viability was assessed in a similar way as described in the “Indirect Method” section. Where more viable cells were observed, the membrane(s) were chosen to proceed to the in vivo experiments.

### 2.5. In Vivo Evaluation

Aiming to choose the best constructs to be used in clinical tests, membranes’ effectiveness on the healing of wounds and skin regeneration/repair was evaluated. To perform this study, in order to minimize the use of animals, only the membrane with the most promising in vitro results was tested: the L-Chit/VP one. Additionally, taking advantage of the gamma-radiation characteristics that allow preparation and sterilization in one single step, no further membrane sterilization procedures were necessary before implantation.

All surgical procedures were performed using aseptic techniques and following all technical and ethical rules mandatory in the use of experimentation animals. All animal studies were approved by the Ethical Committee of Nova Medical School, the institution where the experiments were conducted (45/2017/CEFCM).

**Figure 2:**
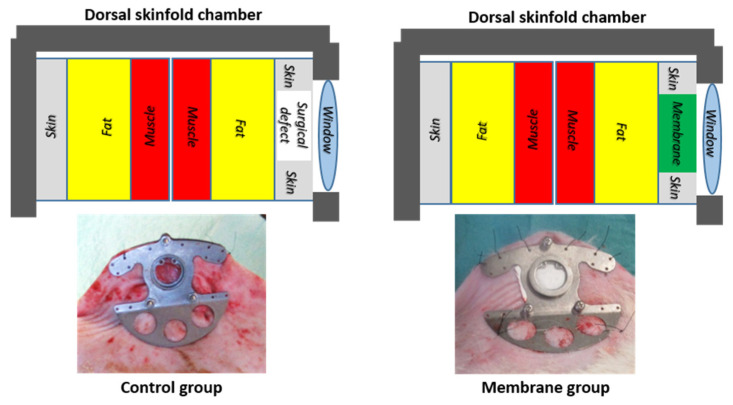
Schematic representation and images of the dorsal skinfold chamber in the rat’s dorsum in control and membrane groups.

The in vivo evaluation was performed in 3–6 months old male Wistar rats with an average weight of 400 g. The animals were subjected to surgical procedure skin excision under general anesthesia, and a dorsal skinfold chamber model of wound repair was used [29]. This model (vd. Figure 2) allows to create a standardized wound, i.e., a surgical defect (3 mm in depth and 10 mm in diameter) on one side of a fold created in integument of the dorsum of the rat and to follow that wound non-invasively for up to six weeks [29]. In this study, two experimental groups were established: the control group, where the wound was secured to the dorsal skinfold chamber and was left to heal spontaneously by secondary intention (*n* = 5); the membrane group, where the wound was covered with the developed membrane (*n* = 10).

For both groups, the following parameters were assessed:

- Daily clinical assessment of rats;
- Wound epithelization area was determined weekly by non-invasive intravital microscopy using a transillumination technique;
- Histological assessment of the ulcer region 3 weeks post-operatively concerning:
  (i) General architecture (hematoxylin-eosin, and Masson’s Trichrome staining—these are common methods for assessing cellular and extracellular composition and spatial distribution, particularly of collagen fibers in human skin [30]);
  (ii) Epithelial growth (immunohistochemical staining for basal undifferentiated keratinocytes (CK5—Rabbit Anti-Human Keratin 5 Monoclonal Antibody (Clone SP178 ^®^Roche Diagnostic);
  (iii) Connective tissue growth (immunohistochemical staining for fibroblasts (Vimentin-Rabbit Anti-Human Vimentin Monoclonal Antibody (Clone SP20) ^®^Roche Diagnostic);
  (iv) Vascular tissue growth (immunohistochemical staining for endothelial cells (CD-31-Rabbit Anti-Human CD31 Monoclonal Antibody (Clone SP164) ^®^Roche Diagnostic [3,4]).

All immunohistochemical protocols were performed on ^®^Roche’s BenchMark ULTRA. Detailed protocol is described in the Supplementary Materials. 

## 3. Results and Discussion

The authors’ goals in this work were to identify and evaluate, in vivo, the most promising biocompatible and biodegradable chitosan-based membranes with improved healing and tissue-regenerating/repair capabilities to be used as skin scaffolds, which resulted from previous work on formulation and preparation methodology optimization. The study focused on membranes’ physical-chemical and biological in vitro characterization and on the in vivo evaluation of wound healing and tissue regeneration using a dorsal skinfold chamber rat model. The use of a dorsal skinfold chamber is an attractive technique for monitoring vascularization and wound healing [29,31]. It is worth mentioning that the hydrophilic character and the crosslinked extension of the developed membranes as a function of their composition and irradiation dose was already studied by FTIR, TGA, and contact angle measurement and published elsewhere [22,23]. 

### 3.1. Membranes’ Characterization

#### 3.1.1. Scanning Electron Microscopy (SEM)

**Figure 3:**
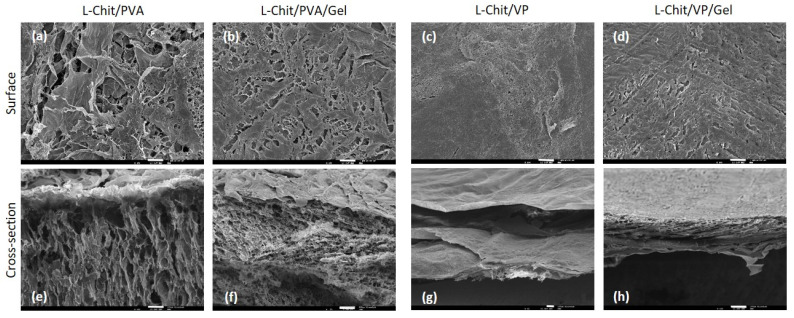
SEM images of the low-Mw chitosan-based membranes γ-irradiated at 10 kGy (scale bar 100 μm and magnification 100×): (**a**–**d**) Membranes’ surface; (**e**–**h**) membranes’ cross-section.

Surface and cross-section SEM images (Figure 3) of the chitosan membranes γ-irradiated at 10 kGy show significant differences depending on membranes’ composition. 

Since the irradiation methodology and radiation dose applied were the same for all the membranes, surface morphology and the inner part are dependent on membranes’ composition. L-Chit/PVA membranes apparently have larger pore size than the L-Chit/VP ones and a sponge-like inner structure (Figure 3a,b,e,f). The L-Chit/VP membrane, on the other hand, displays a heterogeneous layered structure with small sponge-like inner regions (Figure 3c,g). The analysis of the SEM images and membranes’ content suggests that the difference in the inner structure may be related not only with the different composition, but also due to the use of a polymer (PVA) and a monomer (VP) in the different formulations originating a more structured region in L-Chit/PVA membranes. Concerning L-Chit/VP/Gel, we can observe what seems to be a more compact sponge-like structure, probably due to some collapse of a less dense structure compared to the other membranes.

The different morphologies and pore dimensions observed assume particular importance for the intended application since the skin scaffolds must be capable of mimicking the structure and biological functions of the extracellular matrix, providing, in parallel, a good environment for cell attachment, proliferation and differentiation [32]. In this way, if on the one hand a more compact membrane can provide better protection, the existence of greater porosity can facilitate the creation of new tissues and promote an easier vascularization [33]. 

#### 3.1.2. Swelling

**Figure 4:**
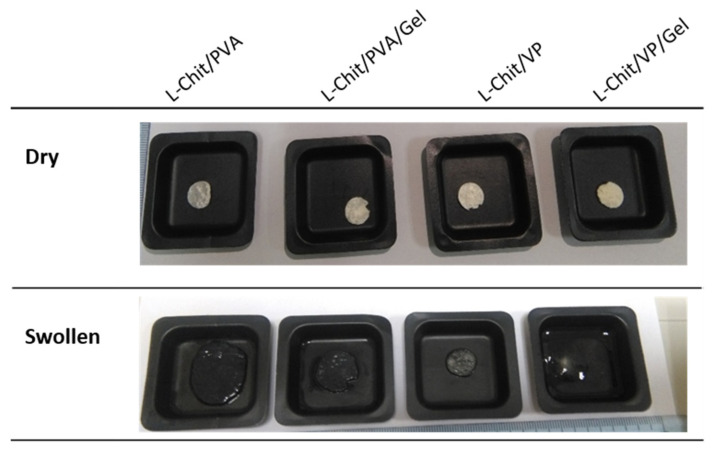
Macroscopic view of low-Mw chitosan-based membranes γ-irradiated at 10 kGy in dry and swollen state after 2 h immersed in distilled water.

**Figure 5:**
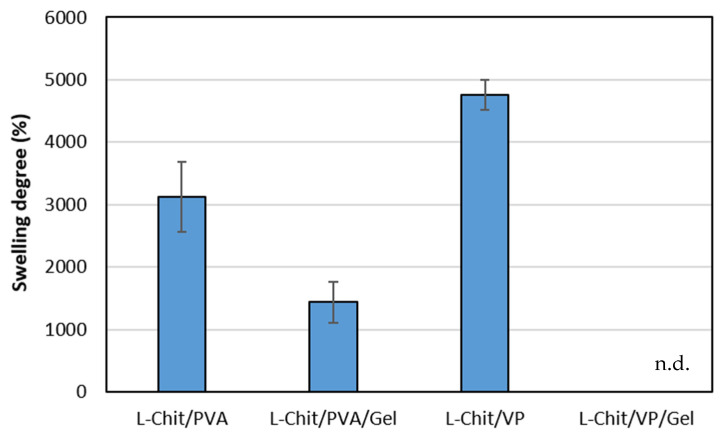
Swelling degree in distilled water of low-Mw chitosan-based membranes γ-irradiated at 10 kGy expressed as mean ± SD (*n* = 3); n.d.: not determined.

As mentioned earlier in Section 2.3.2, swelled chitosan-based membranes gamma-irradiated at 10 kGy were removed from the initial container and placed in another for weighting. With the exception of the L-Chit/VP/Gel membrane that practically dissolved after 2 h immersed in water, all the others had enough structural stability to be handled, albeit with care in order to avoid tearing. This behavior suggests the occurrence of successful γ-induced crosslinking reactions in the majority of the obtained membranes (vd. Figure 4). Simultaneously, an increase in membranes’ volume was observed, which was indicative of their high swelling capacity, as confirmed by quantitative studies depicted in Figure 5.

From Figure 5, it is clear that chitosan-based membranes without gelatin in their composition are the ones that presented the highest swelling degree values. On the other hand, membranes that also incorporate gelatin presented lower swelling values. Particularly, in the case of L-Chit/VP/Gel, membranes turned into a swollen transparent soft matter close to soluble, making it impossible to handle and weight the sample. For that membrane, it was not possible to determine membranes’ swelling degree. The reason for that could be related to gelatin itself, which is a mixture of water-soluble proteins. In this way, the solubilization of gelatin would lead to samples’ weight loss and consequent lower swollen weight. Additionally, these results also seem to indicate that the extension of gelatin radiation degradation was most likely higher than the eventual radiation-induced crosslinking between the other membranes’ components and the gelatin. Additionally, contrary to what macroscopic observation suggested, the L-Chit/VP membrane, which was the one with the lowest volume increase, was the one which presented the highest swelling degree.

The swelling is a consequence of the interaction of the polymeric matrix with the solvent, in this case distilled water, which results in an increase in 3D network and geometry. Thus, for partially crosslinked systems, swelling can be used as a measure of the crosslink density, even though swelling may not be directly related with the increase of samples’ volume. Usually, a highly crosslinked polymer is associated to lower porosity and lower swelling degrees since the available space for solvent adsorption will be lower [34,35]. However, in the case of the obtained membranes, L-Chit/VP is the membrane that apparently presents the lowest surface pore size and the lowest volume increase but the highest swelling degree. One possible explanation is due to the layer inner structure observed. Despite showing lower pore size at the surface, this membrane presents a bigger direct available volume between the different layers, allowing the retention of bigger amounts of solvent without greatly increasing the external volume as observed. Another possible explanation is related with the chitosan content. By having the same content in terms of PVA and VP, and since PVA was added to chitosan solution as a 10% m/v solution and VP as a 98% m/v, for the same final volume, the final content in chitosan in L-Chit/PVA membranes will be lower than the one in L-Chi/VP membranes. In this way, the differences in the swelling degree seem to be related with the chitosan content and its respective extension of crosslinking and not in terms of the hydrophilicity of VP or PVA [36]. However, this high swelling capacity together with the retention of physical stability are key factors for the use in in vivo tests. High swelling values may indicate that body fluids can permeate the membrane and thus facilitate its integration into the existing skin and into the one that is being regenerated.

#### 3.1.3. In Vitro Degradation

**Figure 6:**
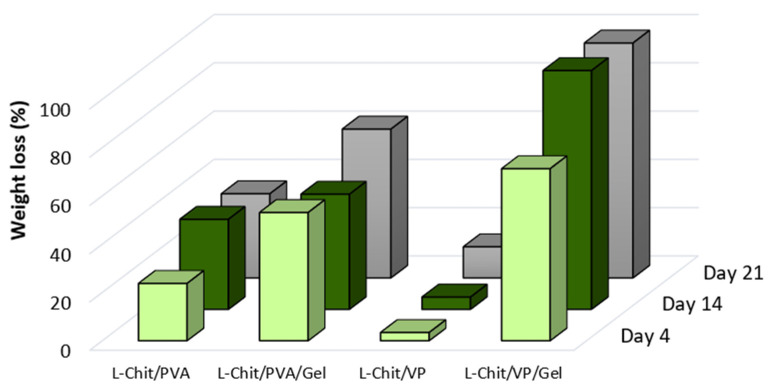
Weight loss of low-Mw chitosan-based membranes γ-irradiated at 10 kGy after 4, 14 and 21 days immersed in saline solution at 36 °C.

After 21 days soaking in 0.9% *m*/*v* NaCl at 36 °C, before drying, only L-Chit/VP retains its initial shape, while all other membranes were very swollen. Additionally, with the exception of L-Chit/VP, a great loss of mass occurred in all samples after drying. Figure 6 presents the weight loss of the irradiated chitosan-based membranes after 4, 14 and 21 days immersed in saline solution at 36 °C.

As the L-Chit/VP membranes end up having a higher content of chitosan when compared with the L-Chit/PVA ones, their degradation rate is slower, making them more stable. This stability is crucial for in vivo use since the scaffold degradation rate must match the tissue regeneration rate to allow the simultaneous growth of cells/tissues while the scaffold is degrading [15]. On the other hand, L-Chit/VP is the membrane that presents the highest swelling in water. Thus, when in the presence of saline solution, it may be possible that there is a higher retention of salt inside the matrix, even though it has been washed (held) in water for 10 min; consequently, when the membrane dries, it shows a smaller decrease in its weight.

It was also observed that after drying, L-Chit/PVA membranes became rigid which, considering the known PVA crosslinking activity, was associated to a high level of membranes crosslinking. On the other hand, L-Chit/VP membranes become smaller than the initial dimensions after drying, which can be attributed to their predominant laminar structure, which became denser. In conclusion, the degradation results suggest that the L-Chit/VP is the most promising membrane to be used as skin scaffold in in vivo tests.

### 3.2. In Vitro Evaluation

#### 3.2.1. Indirect Method

After 4 days growing in the presence of the membranes’ conditioned media, apparently healthy cells were observed in the inverted microscope in all the experimental conditions. No signs of cell death or degradation were observed.

**Figure 7:**
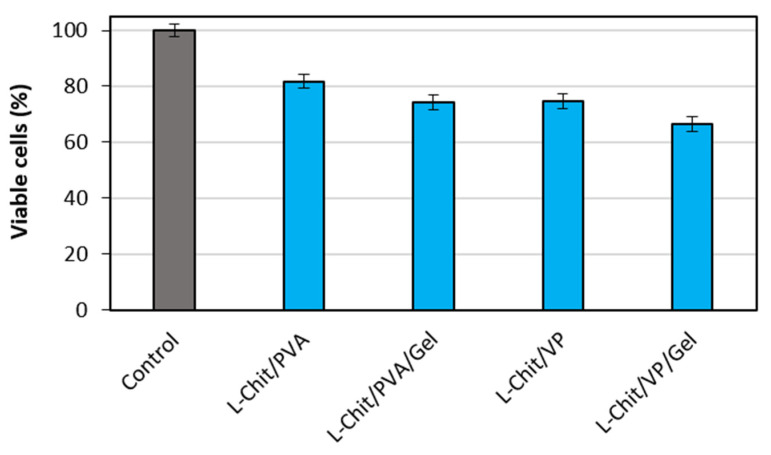
Viable HFFF2 cells growing in conditioned media by the respective membranes (blue bars) as a percentage of control cells (grey bar) growing in non-conditoned medium for 4 days (*n* = 3; mean value ± SD).

The results from the cell viability test (Figure 7) revealed the proliferation of a similar number of HFFF2 cells in all conditions, even though we detected a slightly lower number of cells as compared with the control situation. We therefore conclude that no noticeable cytotoxic effect was exerted on the cells by soluble products released from the membranes to the culture medium.

#### 3.2.2. Direct Method

**Figure 8:**
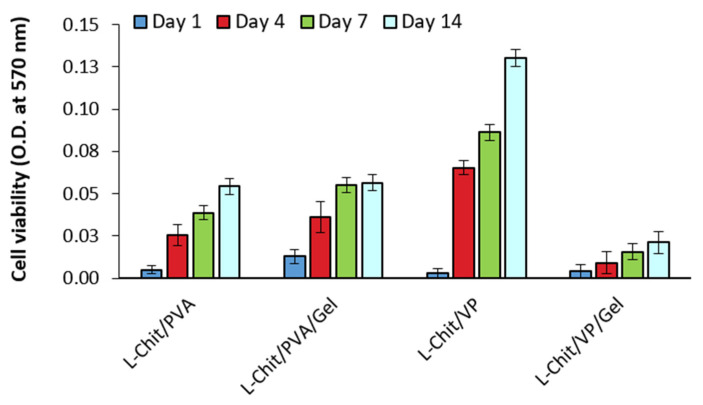
Cellular viability of HFFF2 growing on different substrates on culture days 1, 4, 7 and 14 (*n* = 3; mean value ± SD).

When comparing the number of cells growing on the different membranes over a period of 14 days, using the same cell viability test (Figure 8), we observed that cells were able to proliferate in all conditions. Nevertheless, the membrane that elicited the more efficient HFFF2 growth was the L-Chit/VP one. Therefore, this was the membrane selected to be used in the further in vivo studies; as expected, this membrane is the more suitable one for the colonization of native dermal fibroblasts and, consequently, the repair of the wound.

### 3.3. In Vivo Evaluation

As mentioned before, the L-Chit/VP membrane was the one which led to higher fibroblast proliferation and thus was the one used in the in vivo assays. The daily evaluation of the animals’ clinical status revealed no deaths or signs of animal suffering (change in behavior, anorexia, weight loss, etc.) in either group. No signs of infection were noted either. Good integration of the membrane implant was observed in all cases.

#### 3.3.1. Wound Epithelization

**Figure 9:**
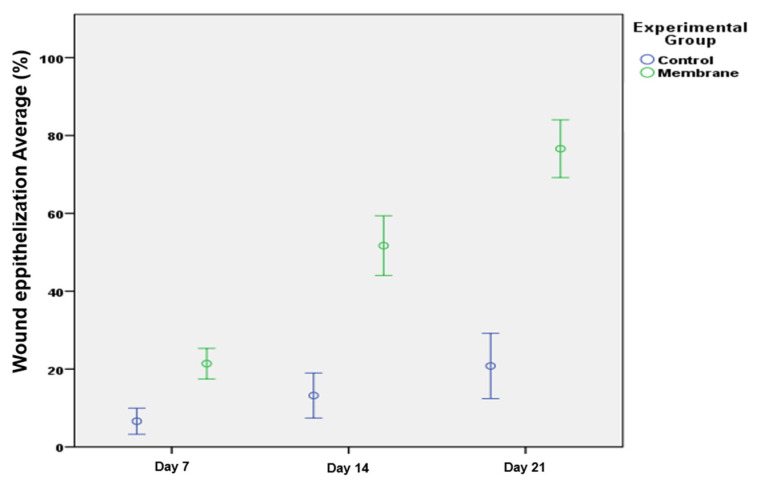
Wound epithelization in the different groups 7, 14 and 21 days postoperatively. Error bars represent 95% confidence intervals.

As can be seen in Figure 9, wound epithelization was faster in the membrane group compared to the negative controls, where the wound was left open for self-healing, filling in and closing up naturally (for each time point *p* < 0.05).

#### 3.3.2. Typical Aspect on the 21st Postoperative Day

**Figure 10:**
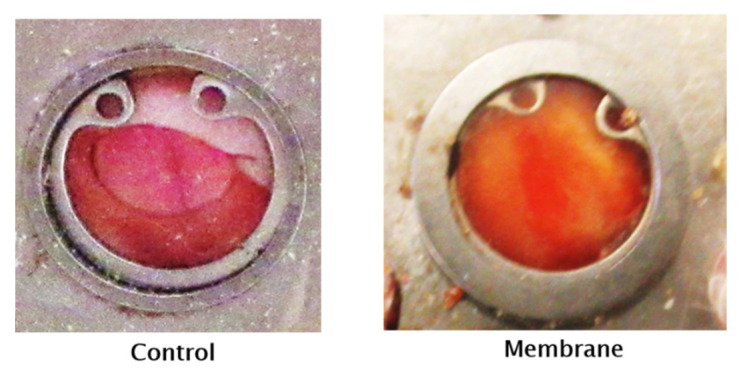
Representative photographs of the dorsal skinfold chamber model in the control and membrane groups on the 21st postoperative day.

The two representative photographs through the dorsal skinfold chamber depicted in Figure 10 illustrate the faster closure of the wound on the 21st postoperative day in the membrane group compared to the control group.

#### 3.3.3. Histological Evaluation

(i) General architecture

To evaluate the general architecture and the presence of collagen, hematoxylin-eosin and Masson’s Trichrome, staining was used since in a standard Masson’s Trichrome procedure collagen is stained green, nuclei are stained dark brown, muscle tissue is stained red and cytoplasm is stained pink [30].

**Figure 11:**
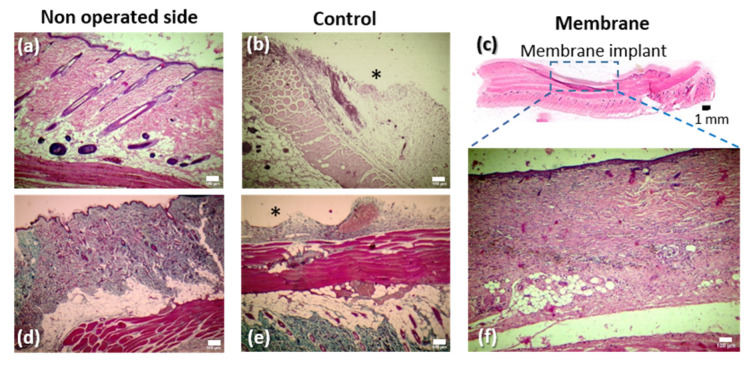
Representative microphotographs depicting the typical histological presentation of the wound in the control and membrane groups, as well of that of the non-operated side of the dorsal skinfold of the rat on the 21st postoperative day. Hematoxylin-eosin stain was used in the upper row of images (**a**,**b**), whereas Masson’s Trichrome was used in the lower row of images (**d**–**f**) (white scale bar = 100 μm;); * stands for granulation tissue; (**c**) illustrates at a low magnification the two layers of the skin in the defect region.

Regarding the general tissue layout in the wound area, a few differences were observed between groups (vd. Figure 11):

- The control group presented a variable extension where skin was absent in the central part of the wound, being replaced by granulation tissue (*).
- The membrane group showed reconstitution of the two layers of the skin. However, the skin in this group presented some differences relative to the normal skin, namely absence of dermal papillae, ridges and skin appendages.

The non-operated side (Figure 11a,d) shows normal integumentary histology on the dorsum of the rat. The control group (excision group) images (Figure 11b,e) present the defect created by the surgical excision of skin and some underlying fat tissue in the operated side of the skinfold on the dorsum of the rat. This region is mostly invaded by granulation tissue (*). The lower part of these photographs shows the non-operated side of the skinfold with its normal architecture. The upper image of the membrane group (Figure 11c) illustrates, at a low magnification, the adequate reconstitution of the two layers of the skin in the defect region.

(ii) Epithelial, connective and vascular tissue growth

Immunochemistry techniques were used to evaluate epithelial, connective and vascular tissue growths.

**Figure 12:**
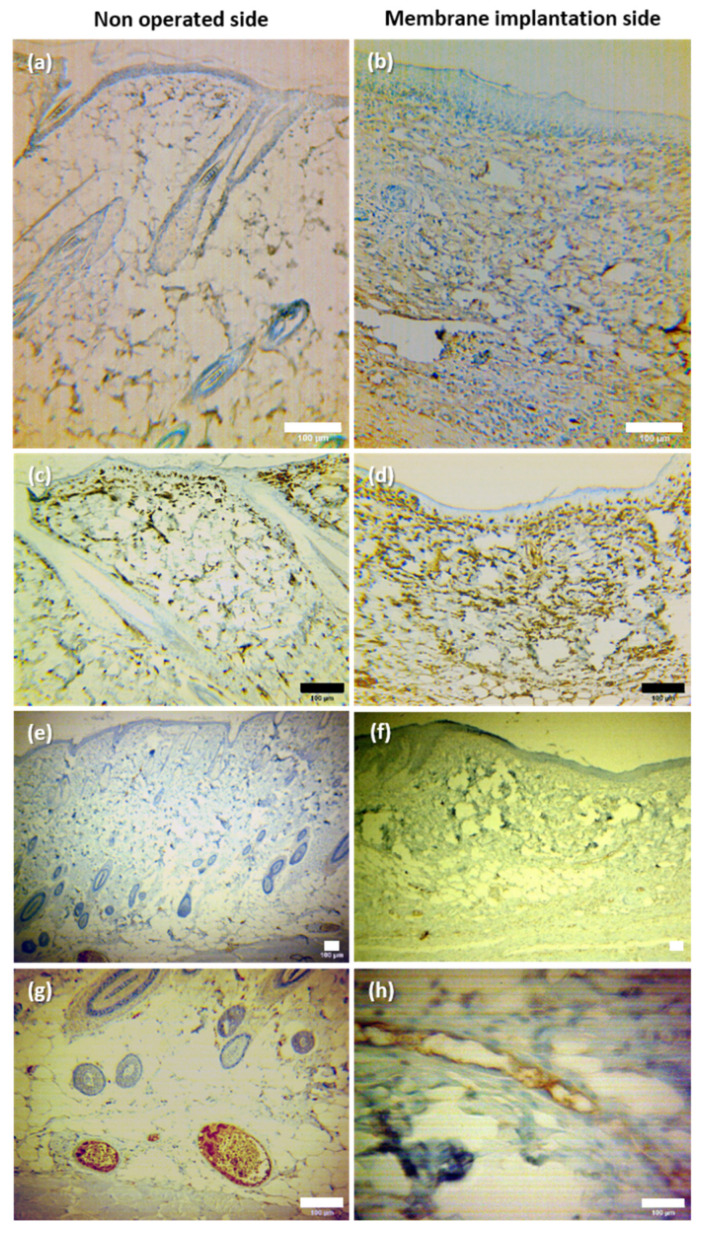
Typical microphotographs of the non-operated side and of the membrane implantation side of the dorsal skinfold chamber after immunohistochemistry staining for: (**a**,**b**) basal undifferentiated keratinocytes (using cytokeratin) 5; (**c**,**d**) fibroblasts (using vimentin); (**e**–**h**) endothelial cells (using CD-31). Scale bar = 100 μm.

In the immunohistochemistry image staining for keratinocytes in Figure 12a,b, it is possible to clearly note that the skin in the membrane implantation side differed from that in the non-operated group due to its greater simplicity and the absence of skin appendages.

In the immunohistochemistry photograph staining for fibroblasts (Figure 12c,d), it was possible to clearly note that the dermis in the matrix group presented a greater number of fibroblasts compared to the contra-lateral side.

The photographs of CD-31 stained sections (Figure 12e–h) show that new vessels could be observed in the membrane implantation site. These vessels were generally smaller than those of the contra-lateral side.

These results show that histologically, the membrane implantation side presented a simpler architecture than the normal tissues. Notwithstanding, keratinocytes, fibroblasts and new vessels were observed in this region. 

It would have been interesting to obtain histological samples of the wound area at different time points during the experiment. However, biopsies to the wound area entail removing and repositioning the dorsal skinfold chamber apparatus and taking a specimen from the wound area. These procedures potentially cause additional discomfort to animals and changes to this region in terms of wound area, wound architecture and wound biology. Furthermore, frequent manipulations of this region increase the risk of infection, which may jeopardize future observations and complicate interpretation of results. Consequently, following what it is generally accepted in the literature, the authors performed histology studies only at the end of the experiment [37].

## 4. Conclusions

The present study showed that the best membrane composition ready to use in animal experiments was the one with a 2% content in low molecular chitosan and 5% in VP, obtained by freeze-drying and γ-irradiation techniques (10 kGy; dose rate = 0.5 kGy·h^−1^). The membrane presents a crosslinked structure which is also microbiologically safe since no additional sterilization process was applied before in vitro and in vivo tests, and no contamination or infection was observed. Membranes’ effectiveness on the healing of wounds and skin regeneration/repair was evaluated by in vivo regeneration experiments in male Wistar rats, and the developed membrane proved to be safe and biocompatible, promoting wound epithelization faster than healing by secondary intention (wound left open for self-healing, filling in and closing up naturally). The membrane group exhibited no signs of infection, a good tissue integration and an accelerated epithelization compared with the control group (excision group) with no membrane. Histologically, the membrane implantation site presented a simpler architectural organization than the normal tissues. Notwithstanding, keratinocytes, fibroblasts and new vessels were observed in the region. Thus, the developed membranes show a promising potential as a new, safe and efficient alloplastic option for skin wound healing. However, additional studies are warranted to document their efficiency and safety in the human clinical setting.

## Data Availability

Not applicable.

## Supplementary Materials

The following supporting information can be downloaded at: https://www.mdpi.com/article/10.3390/pharmaceutics14122736/s1.
