# Supplementary material for: Chitosan-Based Membranes for Skin Wound Repair in a Dorsal Fold Chamber Rat Model
Source: Pharmaceutics. 2022 Dec 7;14(12):2736. doi: 10.3390/pharmaceutics14122736 (PMC9784945; doi:10.3390/pharmaceutics14122736)
Supplement: Supplementary file 1 [file pharmaceutics-14-02736-s001.zip › pharmaceutics-2055654-supplementary.pdf]

*pharmaceutics*

*Article*

# Chitosan-Based Membranes for Skin Wound Repair in a Dorsal Fold Chamber Rat Model

**Maria Helena Casimiro [1,*], Luís M. Ferreira [1,2], Pedro M. P. Santos [1], João P. Leal [2,3], Gabriela Rodrigues [4], Inês Iria [5], Sara Alves [6], Diogo Pais [6] and Diogo Casal [7,*]**

[1] Centro de Ciências e Tecnologias Nucleares (C2TN), Instituto Superior Técnico (IST), Universidade de Lisboa, 2695-066 Bobadela, Portugal; ferreira@ctn.tecnico.ulisboa.pt (L.M.F.); psantos@ctn.tecnico.ulisboa.pt (P.M.P.S.)
[2] Departamento de Engenharia e Ciências Nucleares (DECN), Instituto Superior Técnico (IST), Universidade de Lisboa, 2695-066 Bobadela, Portugal; jpleal@ctn.tecnico.ulisboa.pt
[3] Centro de Química Estrutural (CQE), Instituto Superior Técnico (IST), Universidade de Lisboa, 2695-066 Bobadela, Portugal
[4] Centro de Ecologia, Evolução e Alterações Ambientais (cE3c) & CHANGE—Global Change and Sustainability Institute, Departamento de Biologia Animal, Faculdade de Ciências, Universidade de Lisboa (FCUL), 1749-016 Lisboa, Portugal; mgrodrigues@fc.ul.pt
[5] Instituto de Investigação do Medicamento (iMed ULisboa), Faculdade de Farmácia, Universidade de Lisboa, 1649-003 Lisboa, Portugal; inesraqueliria@gmail.com
[6] Departamento de Anatomia, NOVA Medical School | Faculdade de Ciências Médicas (NMS | FCM), Universidade NOVA de Lisboa, 1169-056 Lisboa, Portugal; saraalves79@hotmail.com (S.A.); diogo.pais@nms.unl.pt (D.P.)
[7] Centro de Estudos de Doenças Crónicas (CEDOC), NOVA Medical School | Faculdade de Ciências Médicas (NMS | FCM), Universidade NOVA de Lisboa, 1150-082 Lisboa, Portugal
* Correspondence: casimiro@ctn.tecnico.ulisboa.pt (M.H.C.); diogo_bogalhao@yahoo.co.uk (D.C.); Tel.: +351-219946045 (M.H.C.); +351-916117315 (D.C.)

**Citation:** Casimiro, M.H.; Ferreira, L.M.; Santos, P.M.P.; Leal, J.P.; Rodrigues, G.; Iria, I.; Alves, S.; Pais, D.; Casal, D. Chitosan-Based Membranes for Skin Wound Repair in a Dorsal Fold Chamber Rat Model. *Pharmaceutics* **2022**, *14*, 2736. https://doi.org/10.3390/pharmaceutics14122736

Academic Editor: Katarína Valachová

Received: 9 November 2022
Accepted: 1 December 2022
Published: 7 December 2022

**Publisher's Note:** MDPI stays neutral with regard to jurisdictional claims in published maps and institutional affiliations.

**Copyright:** © 2022 by the authors. Submitted for possible open access publication under the terms and conditions of the Creative Commons Attribution (CC BY) license (https://creativecommons.org/licenses/by/4.0/).

## S1. CK5 Protocol Summary

**Procedure: U ultraView DAB (v1.02.0018)**
**BenchMark ULTRA IHC/ISH Staining Module**

1 Baking [Selected]
2 Warmup Slide to [72 Deg C], and Incubate for [4 Minutes] (Baking)
3 Deparaffinization [Selected]
4 Warmup Slide to [74 Deg C] from Medium Temperatures (Deparaffinization)
5 Cell Conditioning [Selected]
6 ULTRA Conditioner #1 [Selected]
7 Warmup Slide to [97 Deg C], and Incubate for 8 Minutes (Cell Conditioner #1)
8 20 minutes of ULTRA CC1 [Selected]
9 36 minutes of ULTRA CC1 [Selected]
10 52 minutes of ULTRA CC1 [Selected]
11 Enzyme [Selected]
12 Apply One Drop of [PROTEASE 3] (Enzyme), Apply Coverslip, and Incubate for [8 Minutes]
13 Ab Incubation Temperatures [Selected]
14 Warmup Slide to [39 Deg C], and Incubate for 4 Minutes (Antibody)
15 Titration [Selected]
16 Hand Apply (Primary Antibody), and Incubate for [0 Hr 32 Min]
17 Counterstain [Selected]
18 Apply One Drop of [HEMATOXYLIN] (Counterstain), Apply Coverslip, and Incubate for [4 Minutes]
19 Post Counterstain [Selected]
20 Apply One Drop of [BLUING REAGENT] (Post Counterstain), Apply Coverslip, and Incubate for [4 Minutes]
* one drop is one reagent dispense

CHLC-H.S.JOSE, LISBOA
VSS v12.3.1 Build 18312.1

**S2. Vimentin Protocol Summary**

**Procedure: U ultraView DAB (v1.02.0018)**
**BenchMark ULTRA IHC/ISH Staining Module**

1 Baking [Selected]
2 Warmup Slide to [75 Deg C], and Incubate for [4 Minutes] (Baking)
3 Deparaffinization [Selected]
4 Warmup Slide to [72 Deg C] from Medium Temperatures (Deparaffinization)
5 Extended Depar [Selected]
6 Cell Conditioning [Selected]
7 ULTRA Conditioner #1 [Selected]
8 Warmup Slide to [95 Deg C], and Incubate for 8 Minutes (Cell Conditioner #1)
9 20 minutes of ULTRA CC1 [Selected]
10 36 minutes of ULTRA CC1 [Selected]
11 Ab Incubation Temperatures [Selected]
12 Warmup Slide to [37 Deg C], and Incubate for 4 Minutes (Antibody)
13 Titration [Selected]
14 Hand Apply (Primary Antibody), and Incubate for [0 Hr 16 Min]
15 Counterstain [Selected]
16 Apply One Drop of [HEMATOXYLIN] (Counterstain), Apply Coverslip, and Incubate for [4 Minutes]
17 Post Counterstain [Selected]
18 Apply One Drop of [BLUING REAGENT] (Post Counterstain), Apply Coverslip, and Incubate for [4 Minutes]
* one drop is one reagent dispense
CHLC-H.S.JOSE, LISBOA
VSS v12.3.1 Build 18312.1

**S3. CD31 Protocol Summary**

**Procedure: U ultraView DAB (v1.02.0018)**
**BenchMark ULTRA IHC/ISH Staining Module**

1 Baking [Selected]
2 Warmup Slide to [75 Deg C], and Incubate for [4 Minutes] (Baking)
3 Deparaffinization [Selected]
4 Warmup Slide to [74 Deg C] from Medium Temperatures (Deparaffinization)
5 Extended Depar [Selected]
6 Cell Conditioning [Selected]
7 ULTRA Conditioner #1 [Selected]
8 Warmup Slide to [95 Deg C], and Incubate for 8 Minutes (Cell Conditioner #1)
9 20 minutes of ULTRA CC1 [Selected]
10 36 minutes of ULTRA CC1 [Selected]
11 52 minutes of ULTRA CC1 [Selected]
12 64 minutes of ULTRA CC1 [Selected]
13 Ab Incubation Temperatures [Selected]
14 Warmup Slide to [37 Deg C], and Incubate for 4 Minutes (Antibody)
15 Antibody [Selected]
16 Apply One Drop of [CD31 (JC70)] (Antibody), Apply Coverslip, and Incubate for [0 Hr 32 Min]
17 Counterstain [Selected]

18 Apply One Drop of [HEMATOXYLIN] (Counterstain), Apply Coverslip, and Incubate for [4 Minutes]
19 Post Counterstain [Selected]
20 Apply One Drop of [BLUING REAGENT] (Post Counterstain), Apply Coverslip, and Incubate for [4 Minutes]
* one* one drop is one reagent dispense
CHLC-H.S.JOSE, LISBOA
VSS v12.3.1 Build 18312.1
